# Molecular characterization of hepatitis A virus isolates from environmental and clinical samples in Greece

**DOI:** 10.1186/1743-422X-7-235

**Published:** 2010-09-16

**Authors:** Petros Kokkinos, Panos Ziros, Sevasti Filippidou, Ioannis Mpampounakis, Apostolos Vantarakis

**Affiliations:** 1 Environmental Microbiology Unit, Department of Public Health, School of Medicine, University of Patras, Greece

## Abstract

**Background:**

Hepatitis A virus (HAV) strains detected in environmental and clinical samples were analysed to characterize the genotypes of HAV circulating in Greece. Fifty (50) sewage samples were collected from Patras (South-Western Greece) and Alexandroupolis (North-Eastern Greece) from 2007 until 2009, accordingly. The clinical samples derived from an HAV outbreak involved populations from three neighbouring prefectures of North-Eastern Greece (Xanthi, Rodopi, and Evros). HAV particles were detected by nested RT-PCR, using a previously validated set of primers to amplify a 290-bp fragment encompassing the 5'-NTR. Positive HAV samples were confirmed by sequencing of the PCR product. To determine the relatedness between the different isolated sequences, a phylogenetic tree was constructed.

**Results:**

Results showed a 100% prevalence of genotype I, and particularly subgenotype IA. The analyzed HAV strains were closely related between them with the percentage of nucleotide identity ranging between 96% and 100%.

**Conclusions:**

The study revealed the major prevalence of circulating strains of IA genotype in Greece and underlined the usefulness of molecular methods for the detection and typing of viruses in both environmental and clinical samples. The present study is, to our knowledge, the first in Greece to depict the simultaneous molecular characterization of HAV strains isolated from both clinical and environmental samples.

## Background

The Hepatitis A virus (HAV) is responsible for around half the cases of hepatitis diagnosed worldwide and is recognized currently as one of the most important human food-borne pathogens, as it is the cause of most outbreaks reported in the Western world. It is not possible to distinguish HAV strains by serotyping, but seven genotypes can be differentiated with molecular methods [1]. HAV infection is present in a worldwide distribution, although its endemicity varies significantly at both international and national levels [2]. Genotype I is the most prevalent genotype, comprising at least 80.0% of circulating human strains. The geographical origin of the genotypes correlates with the virus isolates. Sub-genotype IA has been defined as the major HAV in the population in America. In Europe, a more heterogenous pattern is observed with co-circulation of genotypes IA and IB [3]. The detection of HAV is important for diagnosis and epidemiological studies of hepatitis A. Because of the slow and non-cytopathic replication of wild-type (wt) HAV strains, detection of HAV normally utilizes reverse transcription (RT) coupled to polymerase chain reaction (PCR) [4].

An epidemiological shift, from high to low prevalence, has been observed in recent decades in the countries of Southern Europe, including Greece. Consequently, the Mediterranean basin as a whole should no longer be considered as an endemic area [5,6]. Studies, conducted two decades ago in Greece and referenced in more recent reports, albeit with limited sample sizes, indicated a significant reduction in the incidence of hepatitis A probably due to the improvement in socioeconomic conditions [2,7,8]. The last reported HAV outbreak in Greece involved Roma populations in three Prefectures located in the northeast of the country [9]. Current available national data regarding the disease burden of hepatitis A in Greece are thin due to the very limited recent seroepidemiological studies and to the significant underreporting of infection rates. The latest national cross-sectional seroprevalence survey indicated that hepatitis A infection is prevalent in Greece. The National Advisory Committee for Immunization concluded that the hepatitis A vaccine should be included in the Greek National Immunization Program (GNIP) as of January 2008 [8]. To our knowledge, only one of the very few studies performed in the last decade in Greece has compared clinical and environmental HAV strains [10].

The aim of the present study was to correlate HAV isolates from clinical and environmental samples by applying molecular methods in order to reveal the prevalence of genotypes of HAV in Greece. HAV strains from environmental sewage samples, analysed over a 2-year period (2007-2009), were collected from the cities of Patras and Alexandroupolis. Clinical HAV strains were collected during a major HAV outbreak among patients with acute hepatitis, from the hospitals of the cities of Alexandroupolis, Komotini and Xanthi.

## Methods

### Sewage samples

A total of one hundred (100) sewage samples were collected from the biological treatment plants of two large Greek towns, Patras (SW Greece) and Alexandroupolis (NE Greece), from 2007 until 2009. Samples were collected at the entry-point of the Patras' biological treatment plant, which receives sewage from a population of about 250,000 inhabitants. Sewage samples were also collected from both the entry and exit-points of the treatment plant of Alexandroupolis, a city of approximately 50,000 inhabitants. Sewage sampling, concentration and HAV detection were performed according to previously described protocols [10,11].

### Human serum samples

Serum samples from hospitalized HAV outbreak cases were collected and analysed for HAV as previously described [9]. A total of 124 cases were diagnosed with hepatitis A on the basis of their positivity for the hepatitis A IgM antibody (IgM anti-HAV) by hospital laboratories between July and November 2007. HAV isolates were sequenced from eight (8) sera samples from hospitalized patients with acute hepatitis during the outbreak [9].

### Nucleic acid extraction and enzymatic amplification

Viral nucleic acids were extracted using the QIAamp Viral RNA mini-kit (Qiagen), in line with the manufacturer's instructions. Reverse transcription polymerase chain reaction (RT-PCR) and nested PCR techniques were used for the detection of HAV, according to previously published protocols [10]. A 290-bp fragment encompassing the 5'-NTR part was amplified with the same protocol from all samples (environmental and clinical) by employing previously validated primer sets [9].

### Sequencing and analysis of viral genomes

All positive samples (environmental and clinical) were confirmed by sequencing of the PCR product. The purified PCR products of the clinical HAV strains were sequenced by Lark Technologies (Essex, UK), and the environmental strains by VBC-Biotech (Austria). The nucleotide sequences obtained were analyzed by BLAST N program at the NIH website (NCBI, National Centre for Technology Control, NIH, USA), and were compared with each other and with other published sequences deposited in the GenBank database. Multiple alignments were performed using Clustal W2 software http://www.ebi.ac.uk. The neighbour-joining method was applied for the phylogenetic tree analysis, the reliability of which was assessed by bootstrap resampling (1,000 pseudoreplicates), using MEGA 4.0.2 software. The HAV genotype was determined by comparing the different sequences of the Greek strains included in the phylogenetic analysis with the reference sequences of different HAV genotypes.

## Results

### Presence of HAV in environmental and clinical samples

HAV was detected in four sewage samples (4/50, 8%) collected from the inlet of the biological treatment plant of Patras and in one sample (1/50, 2%) collected from the entry-point of the treatment plant of Alexandroupolis. Sera from eight hospitalized patients with acute hepatitis were collected and analyzed for the detection of HAV genome [9].

### Sequence analysis of HAV RNA genomes

**Figure 1:**
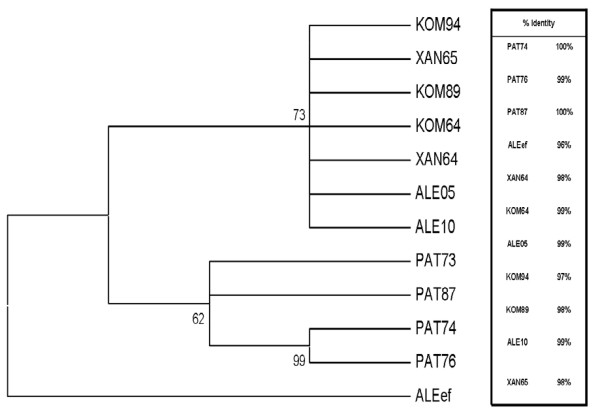
**Phylogenetic tree depicting the relationship between the clinical and the environmental HAV strains of the present study**. Numbers under branches are bootstrap percentage values, calculated from 1,000 bootstrap replicates. Abbreviations are: PAT74, PAT76, PAT87 (sewage samples from the Patras biological treatment plant), ALEef (sewage sample from the Alexandroupolis treatment plant), KOM94-KOM89-KOM64, XAN64-XAN65 and ALE05- ALE10 (clinical strains from the cities of Komotini, Xanthi and Alexandroupolis, respectively). The % nucleotide identity of the nucleotide sequence of PAT73 isolate with the sequences of the other HAV strains of the study is shown on the right.

**Figure 2:**
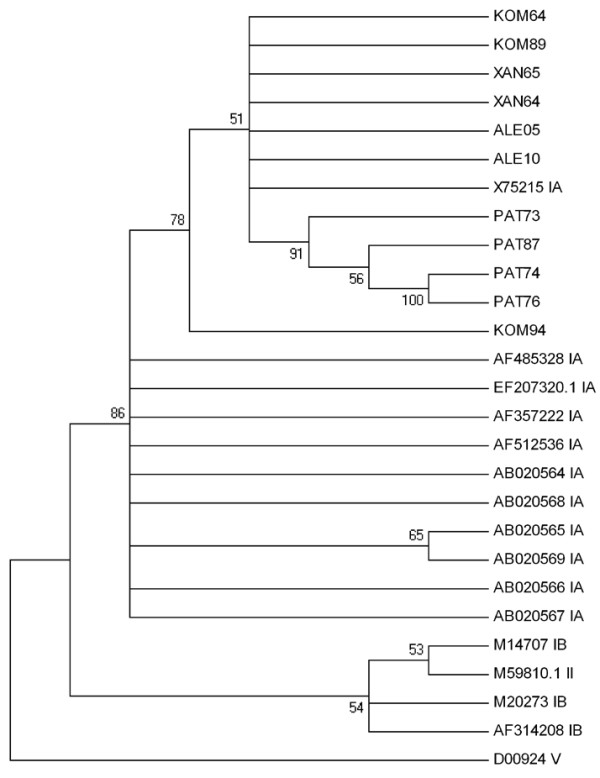
**Phylogenetic tree depicting the relationship between the clinical and the environmental HAV strains of the present study in relation to other isolates retrieved from the GenBank database**. Numbers under branches are bootstrap percentage values, calculated from 1,000 bootstrap replicates. GenBank accession numbers of the reference sequences along with the corresponding genotypes or sub-genotypes are included in the phylogram.

Sequence analysis of the nested PCR products of 5'NTR region, showed high degree of identity among environmental and clinical samples. Comparison of the nucleotide sequence of PAT73 (isolate from sewage of Patras biological treatment plant) with the sequences of the other sewage samples from Patras (PAT74, PAT76, PAT87), the sewage sample from Alexandroupoli (ALEef) and the clinical strains from the cities of Komotini (KOM94, KOM89, KOM64), Xanthi (XAN64, XAN65) and Alexandroupoli (ALE 05, ALE10), showed 96%-100% similarity (Figure 1). This close relationship was confirmed by the phylogenetic analysis, as shown in the phylogenetic trees (Figures 1,2). Comparative analysis of environmental and clinical isolates in our study with other reference isolates (GenBank accession numbers included in the tree) confirms the presence of HAV strains belonging only to genotype IA.

## Discussion

The hepatitis A virus still represents a major public health problem. HAV is a significant cause of morbidity and socioeconomic losses in many parts of the world, while it can result in fulminant hepatitis and death, albeit rarely [12]. HAV has been reported to have an inherently more stable molecular structure than other picornaviruses and thus is characterised by high resistance to the environment and is able to persist for extended periods on environmental surfaces [2]. The incidence of HAV infection varies considerably among and within countries [1]. In the present study, HAV subgenotype IA was detected in sewage samples collected from the biological treatment plants of two urban cities (Patras and Alexandroupolis) in northern and southern Greece. Different patterns of HAV endemicity have been detected in the cities of Cairo and Barcelona, while the circulating strains as characterized by the analysis of sewage samples were genotype IB [6]. A similar study, performed in order to depict HAV strains in Barcelona, from both environmental and clinical samples, revealed a 95% prevalence of genotype I, with nearly 50% being either subgenotype IA or subgenotype IB. Interestingly, in the year 2000, although the number of cases of hepatitis A has been estimated to be less than 15-30 per 100000 habitants, 80% of urban sewage samples studied showed the presence of HAV. This was attributed to the fact that faecal shedding of HAV can last for months after the resolution of symptoms, and patients could be a source of further virus spreading within the community [11]. Analysis of the environmental and clinical isolates of our study showed the presence of HAV strains belonging to genotype IA. Subtype IA appears to be responsible for the majority of hepatitis A cases worldwide, whereas subtype IB viruses have been found in the Mediterranean region [1]. A study of molecular analysis of HAV isolates in Albania has shown that the unique genotype present in Albania is genotype IA [13]. In another study in Albania, only genotype IA was characterized in all the analysed samples of sewage and sera of patients involved in an HAV outbreak [14]. A study from another Mediterranean country, Tunisia, showed that all Tunisian strains belonged to genotype I with a greater presence of sub-genotype IA (98%) and 2% of sub-genotype IB [15].

The hepatitis A vaccine is included in the Greek National Immunization Program (GNIP) and universal vaccination is recommended for all children over 12 months of age [8]. With regards to hepatitis viruses, moving populations such as Roma and refugees constitute special target groups of any population coverage program, as the underlying unfavourable living conditions could facilitate the spread of the infections [12]. Over the past two decades there has been a significant increase in immigration from Eastern Europe and the Balkan countries to Greece. As a result, immigrants of these countries of origin currently comprise 10% of the Greek population [8]. Moreover, due to recent large immigration flows from North Africa and Asia, through Turkey, to Greece, a new epidemiological pattern may emerge in the near future. Data from the occurrence of viruses in sewage may provide an overview of the epidemiology of viral infections circulating in the community, and at the same time reveal the occurrence of asymptomatic infections in the population [6].

## Conclusions

In conclusion, this study - the first in Greece to depict the simultaneous molecular characterization of HAV strains isolated from both clinical and environmental samples - revealed the major prevalence of circulating strains of IA genotype in Greece. Further environmental surveillance could be used in order to enrich the poor existing clinical data from Greece and evaluate the prevalence of HAV in the environment as well as in the community.

Environmental surveillance could prove to be a valuable strategy in the study of prevalence and of the incidence of various pathogens, especially when there is a lack of sufficient clinical data. This lack is mainly due to the fact that most infections develop asymptomatically in children and to the problematic reporting of hepatitis A cases through the surveillance system in Greece.

## Competing interests

The authors declare that they have no competing interests.

## Authors' contributions

PK carried out the sequence alignments, constructed the phylogenetic trees and participated in the writing of the manuscript. PZ participated in the molecular analyses and helped to draft the manuscript. SF and IM collected the samples and participated in the viral concentration, nucleic acids extraction and nested PCRs. AV was responsible for setting up and coordinating the study, and drafted the manuscript. All authors read and approved the final manuscript.

## References

[B1] Sánchez G Populaire S Butot S Putallaz T Joosten H Detection and differentiation of human hepatitis A strains by commercial quantitative real-time RT-PCR tests J Virol Methods 2006 132 160 165 10.1016/j.jviromet.2005.10.010 16280175

[B2] Dounias G Rachiotis G Prevalence of hepatitis A virus infection among municipal solid-waste workers Int J Clin Pract 2006 60 1432 1436 10.1111/j.1742-1241.2006.00845.x 16669822

[B3] Rodrigues L Pista A Oliveira A Agua-Doce I Manita C Paixão MT Molecular epidemiology of hepatitis A virus in a group of Portuguese citizens living in Lisbon area J Med Virol 2007 79 483 487 10.1002/jmv.20851 17387747

[B4] Kwon OS Byun KS Yeon JE Park SH Kim JS Kim JH Bak YT Kim JH Lee CH Detection of hepatitis A viral RNA in sera of patients with acute hepatitis A J Gastroenterol Hepatol 2000 15 1043 1047 10.1046/j.1440-1746.2000.02291.x 11059935

[B5] Arvanitidou M Mamassi P Vayona A Epidemiological evidence for vaccinating wastewater treatment plant workers against hepatitis A and hepatitis B virus Eur J Epidemiol 2004 19 259 262 10.1023/B:EJEP.0000020444.64546.3b 15117120

[B6] Pintó RM Alegre D Domínguez A El-Senousy WM Sánchez G Villena C Costafreda MI Aragonès L Bosch A Hepatitis A virus in urban sewage from two Mediterranean countries Epidemiol Infect 2007 135 270 273 10.1017/S0950268806006753 16817987 PMC2870566

[B7] Mazokopakis E Vlachonikolis J Philalithis A Lionis C Seroprevalence of hepatitis A, B and C markers in Greek warship personnel Eur J Epidemiol 2000 16 1069 1072 10.1023/A:1010857128629 11421478

[B8] Kyrka A Tragiannidis A Cassimos D Pantelaki K Tzoufi M Mavrokosta M Pedeli X Athanassiadou F Hatzimichael A Konstantopoulos A Kafetzis D Papaevangelou V Seroepidemiology of hepatitis A among Greek children indicates that the virus is still prevalent: Implications for universal vaccination J Med Virol 2009 81 582 587 10.1002/jmv.21434 19235841

[B9] Vantarakis A Nearxou A Pagonidis D Melegos F Seretidis J Kokkinos P Zarkadis I Parasidis T Alamanos Y An outbreak of hepatitis A in Roma populations living in three prefectures in Greece Epidemiol Infect 2010 138 1025 1031 10.1017/S0950268809991257 19941688

[B10] Kokkinos P Filippidou S Karlou K Vantarakis A Molecular Typing of Enteroviruses, Adenoviruses, and Hepatitis A Viruses in Untreated and Treated Sewage of a Biological Treatment Plant in Greece Food Environ Virol 2010 2 89 96 10.1007/s12560-010-9036-3

[B11] Pina S Buti M Jardí R Clemente-Casares P Jofre J Girones R Genetic analysis of hepatitis A virus strains recovered from the environment and from patients with acute hepatitis J Gen Virol 2001 82 2955 2963 11714971 10.1099/0022-1317-82-12-2955

[B12] Michos A Terzidis A Kalampoki V Pantelakis K Spanos T Petridou ET Seroprevalence and risk factors for hepatitis A, B, and C among Roma and non-Roma children in a deprived area of Athens, Greece J Med Virol 2008 80 791 797 10.1002/jmv.21134 18360892

[B13] Gabrieli R Sanchez G Macaluso A Cenko F Bino S Palombi L Buonomo E Pinto RM Bosch A Divizia M Hepatitis in Albanian children: Molecular analysis of hepatitis A virus isolates J Med Virol 2004 72 533 537 10.1002/jmv.20028 14981754

[B14] Divizia M Gabrieli R Macaluso A Bagnato B Palombi L Buonomo E Cenko F Leno L Bino S Basha A Panà A Nucleotide correlation between HAV isolates from human patients and environmental samples J Med Virol 2005 75 8 12 10.1002/jmv.20229 15543594

[B15] Gharbi-Khelifi H Sdiri K Harrath R Fki L Hakim H Berthomé M Billaudel S Ferre V Aouni M Genetic analysis of HAV strains in Tunisia reveals two new antigenic variants Virus Genes 2007 35 2 155 159 10.1007/s11262-007-0093-0 17393293

